# Don't words come easy? A psychophysical exploration of word superiority

**DOI:** 10.3389/fnhum.2013.00519

**Published:** 2013-09-04

**Authors:** Randi Starrfelt, Anders Petersen, Signe Vangkilde

**Affiliations:** Department of Psychology, Center for Visual Cognition, University of CopenhagenCopenhagen, Denmark

**Keywords:** reading, word processing, Theory of Visual Attention (TVA), word superiority effect, visual processing speed, visual short term memory

## Abstract

Words are made of letters, and yet sometimes it is easier to identify a word than a single letter. This *word superiority effect* (WSE) has been observed when written stimuli are presented very briefly or degraded by visual noise. We compare performance with letters and words in three experiments, to explore the extents and limits of the WSE. Using a carefully controlled list of three letter words, we show that a WSE can be revealed in vocal reaction times even to undegraded stimuli. With a novel combination of psychophysics and mathematical modeling, we further show that the typical WSE is specifically reflected in perceptual processing speed: single words are simply processed faster than single letters. Intriguingly, when multiple stimuli are presented simultaneously, letters are perceived more easily than words, and this is reflected both in perceptual processing speed and visual short term memory (VSTM) capacity. So, even if single words come easy, there is a limit to the WSE.

## Introduction

The popular notion that we see words as images or objects is reflected in the widely held belief (aided by an email epidemic some years back) that as long as the first and last letters are correctly positioned it “deosn't mttaer in waht oredr the ltteers in a wrod are (…) bcuseae the huamn mnid deos not raed ervey lteter by istlef, but the wrod as a wlohe.” Contemplating the time it takes to even read this misspelt sentence, its claim is obviously not entirely correct (see Grainger and Whitney, 2004). Single letter processing has been shown to be of utmost importance for word reading (e.g., Pelli et al., 2003; Grainger and Dufau, 2012), but the relationship between letter and word processing is complex and yet underspecified.

The *word superiority effect* (WSE) refers to the observation that when written stimuli are degraded by noise or brief presentation, letters in words are reported more accurately than single letters and letters embedded in non-words. This effect has been studied using different tasks, stimuli, and masking conditions (see e.g., Johnston, 1981). In the classical Reicher-Wheeler paradigm, words, non-words, and/or single letters are presented for a single, brief exposure duration and then masked, followed by a forced choice decision about which of two letters was present (Reicher, 1969; Wheeler, 1970). The finding of a superior performance with words in such experiments was one of the driving forces in the development of the Interactive Activation Model of visual word processing (IAM; McClelland and Rumelhart, 1981). In this model, word recognition is achieved through processing on three interactive levels, where activation on higher levels (i.e., word representations) may strengthen or inhibit activations on the letter level. These feedback connections were suggested to be important in explaining the WSE, as this top–down activation of letters renders them more active, than does bottom–up activation alone (which is more likely to be the case when the stimulus is a single letter or a string of unrelated letters).

Visual word processing, and its cerebral substrate, has been intensively investigated in both brain injured and normal participants following the suggestion that a region in the left occipito-temporal cortex—the Visual Word Form Area—may be specialized for the processing of written letter strings (Cohen et al., 2000, 2002). The relationship between the fast, parallel processing of words in canonical format (central presentation of same case words), and effects of word length on processing when stimuli are in some way degraded or distorted (cAsE MiXinG, s p a c i n g, or vertically tilted) has also been studied within this framework, and it has been suggested that the VWFA and the ventral visual stream contribute significantly to processing of words in a canonical format, while attentional mechanisms, relying more on the dorsal stream may a role when words in distorted formats are processed (Vinckier et al., 2007; Cohen et al., 2008). Although the precise role of the VWFA is highly debated (Dehaene and Cohen, 2011; Price and Devlin, 2011), there is general agreement that processing in the ventral visual stream is important for fast and efficient visual word processing. How single letters are treated by these systems has been less thoroughly investigated, but it may rely on processing in slightly different regions than words and letter strings (Flowers et al., 2004; James et al., 2005). Of particular interest for the current study is the point that “the processing of non-pronounceable letter strings cannot be assumed to be equivalent to single-letter perception” (James et al., 2005, p. 452).

This relates to a conceptual distinction made in the cognitive research on word, non-word, and letter processing, between the classical WSE (defined as superior report of letters in words over non-words) and the *word-letter phenomenon* (defined as superior report of letters in words compared to single letters, Jordan and Bevan, 1994). Both phenomena obviously reflect a “word superiority” in processing, and although the word-nonword effect has received the most attention in the experimental literature, the word-letter effect may be the most thought-provoking one: Even if words consist of single letters, and even if there are strong indications that individual letters must be processed for a word to be recognized, words enjoy a processing advantage compared with single letters. Following the IAM (McClelland and Rumelhart, 1981), most will agree that the word advantage is due to top–down effects on word recognition, that are absent or smaller for single letters. It is not clear, however, if this processing advantage may affect the threshold for visual processing of words and letters, or whether it is mainly reflected in the perceptual processing speed. It is also not known how word and letter processing may differ at the level of visual short term memory (VSTM). Can words be encoded as units or wholes in the sense that they are treated like entities in VSTM?

**Figure 1:**
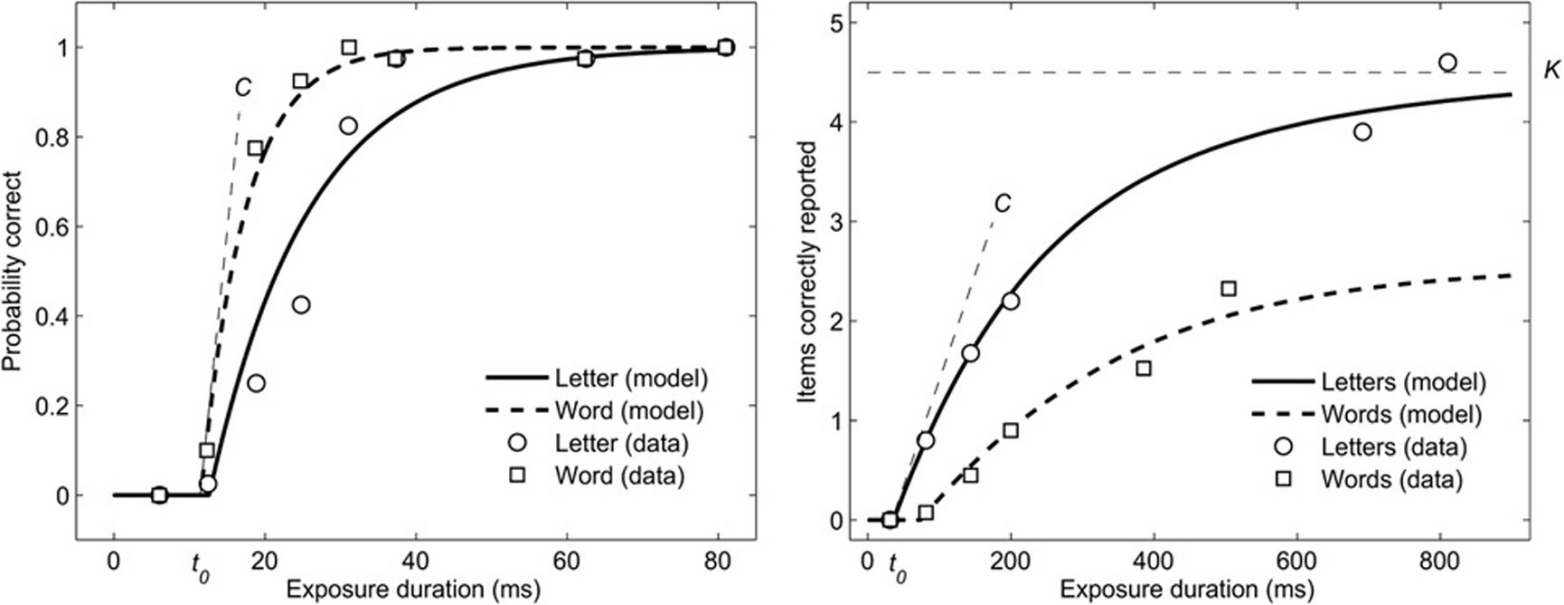
**Illustration of observed data and model fit for letter and word processing in a single subject in Experiments 2 (left) and 3**.

In the current study, we investigate these questions using classical psychophysical paradigms with words and letters as stimuli, and methods based on a Theory of Visual Attention (TVA; Bundesen, 1990). TVA is a theoretical framework for understanding and investigating attentional effects at the behavioral (e.g., Peers et al., 2005; Starrfelt et al., 2009; Vangkilde et al., 2011) and neurophysiological level (Bundesen et al., 2005). TVA-based experiments employ unspeeded, accuracy-based measures of perception and attention, and use computational modeling to derive several attentional parameters unconfounded by response times, from one single task. We focus on three of these parameters in the experiments reported here: (1) *t*_0_, the threshold of conscious perception measured in milliseconds; (2) *C*, the speed of visual processing measured in items processed per second; and (3) *K*, the capacity of VSTM measured in number of items. The parameters are illustrated in Figure 1, right panel. Parameters *C* and *t*_0_ can be estimated both in tasks presenting a single stimulus, and in paradigms with multiple stimuli (whole report).

This study contains three experiments, each including both letter and word stimuli. The first, a computerized naming task, was used to familiarize subjects with the stimuli. In the second experiment, we compared performance with single words and letters at a range of exposure durations. This allowed us to investigate whether the WSE was present in a task where stimuli were to be reported (in contrast to the traditional forced choice tasks), and if so, whether the WSE is reflected either in the threshold for conscious processing (*t*_0_) or the perceptual processing speed (*C*), or both. In the third experiment we used a classical whole report paradigm with multiple stimuli, to estimate the capacity of VSTM (i.e., the *K*-value) for words and single letters. The speed *C* and threshold *t*_0_ were also estimated in the whole report paradigm,

## Materials and methods

All experiments were conducted in a semi-darkened room, and subjects were seated ~100 cm from a 19″ CRT monitor running at 160 Hz.

### Subjects

Twenty-one bachelor students (six male; mean age 23, range 19–36) at the University of Copenhagen participated in this study for course credits. All provided written, informed consent.

### Stimuli and masks

The stimuli were the same in all three experiments and were presented in lower case Arial font (point size 40) in white on a black background. The order of tasks and stimulus conditions was counterbalanced across subjects. The letter-condition featured 25 letters of the alphabet (*w* excluded) with the average letter subtending 0.52° (width; range 0.11–0.92) by 0.83° (height; range 0.69–0.97) of visual angle. For the word-condition, 25 high-frequency, three-letter words were chosen so they could not be predicted by identifying only one letter of the word (see Appendix for a list of stimuli). A printed list of the stimuli was present during all experiments, for easy reference. The average word subtended 1.92° (width; range 1.32–2.41) by 0.99° (height; range 0.69–1.20) of visual angle. Masks were rectangular white-on-black pattern masks (2.46° by 2.12° of visual angle) constructed of letter fragments, thus covering both letters and words completely.

### Mathematical modeling

The results from Experiments 2 and 3 were analyzed using Bundesen's theory of visual attention (TVA; Bundesen, 1990). According to TVA, stimuli in the visual field compete in a race for access to a limited visual short-term store of *K* items. Specifically, the speed at which a stimulus *x* in the visual field races for access to VSTM is given by,

(1) $$v_{x}=C\frac{w_{x}}{\sum_{z\;\in \;S}w_{z}}$$

where *C* is the overall speed of visual processing and *w_x_* is the attentional weight of stimulus *x* which is divided by the sum of attentional weights across all stimuli in the visual field, *S*. In other words, the competition for access to VSTM is represented by the attribution of attentional weights such that a stimulus with a high weight will be processed faster (i.e., have a higher probability of being represented in VSTM) than a stimulus with a low weight.

In the special case in which only a single stimulus is presented in the visual field *v_x_* = C (i.e., no competition) and the probability that the stimulus gets represented in VSTM is given by

(2) $$p=1-e^{-v_{x}\left(\tau \;-\;t_{0}\right)}\text{for }\tau >t_{0}$$

where τ is the exposure duration of the stimulus and *t_0_* is the threshold of conscious perception. That is, if the exposure duration of the stimulus is shorter than *t_0_* the probability that the stimulus will be represented in VSTM is zero. However, if the exposure duration is longer than *t_0_* the probability will follow an exponential function (see Figure 1, right panel, for two examples).

In a single stimulus experiment attentional weights and *K* cannot be estimated resulting in a simple model with only two free parameters, *C* and *t_0_*. However, with larger display sizes the complexity of the model increases as does the number of free parameters (see Dyrholm et al., 2011, for a full specification of the model). In Experiment 3, we used a display size of six stimuli resulting in a model with 13 free parameters. Five parameters were used to characterize a probability distribution of the storage capacity of VSTM. Hence the *K*-value reported in the result section is the expected *K* given a particular probability distribution for each individual participant. Another five parameters were used to estimate the attentional weights (*w*-values) at each of the six stimulus locations (one attentional weight was fixed at a value of 1). The remaining three free parameter were used to estimate the threshold of conscious perception, *t_0_*; the speed of visual processing, *C*; and the sensory decay in the unmasked trials. In both Experiments 2 and 3, the individual data were fitted by an improved maximum likelihood fitting procedure using the LibTVA toolbox for MatLab (Dyrholm et al., 2011).

### Experiment 1. stimulus familiarisation

Experiment 1 was a computerized naming task, used to familiarize subjects with the stimuli employed in Experiments 2 and 3. Half the subjects (*n* = 11) performed the letter task first. Stimuli were randomly selected and presented at the center of the screen with an inter-trial interval of 1 s from response to the next stimulus. Subjects were instructed to name the stimuli as quickly as possible, without making errors, and reaction times (RTs) were measured using a voice key. The letter and word conditions included 50 and 100 trials, respectively, and 10 practice trials were included in each condition. RTs below 200 ms and above 900 ms were considered voice key errors and were removed from the data. On average 5.6% (*SD* = 5) of the letter trials and 2.4% (*SD* = 2.7) of the word trials were removed.

### Experiment 2. single item report

Experiment 2 tested identification of single stimuli flashed briefly at the center of the screen. Letters and words were presented in separate blocks of 160 trials. In total, subjects ran 320 trials per condition, and the first and second blocks for each stimulus type were preceded by 30 and 15 practice trials, respectively. In each trial, a single stimulus was chosen randomly and presented for one of eight exposure durations (6–80 ms, randomly intermixed). The stimulus was terminated by a pattern mask shown for 500 ms. Participants were instructed to make an unspeeded report of the stimulus, if they were “fairly certain” of its identity. Responses were recorded by the experimenter. To ensure foveal presentation, participants were required to focus on a centrally placed cross and then initiate the trial by pressing the right mousebutton.

The analysis first compared the proportions of correct responses for the different exposure durations for the two stimulus conditions. Then, participants' performance was modeled individually by TVA (see section Mathematical Modeling for details). This resulted in separate parameter estimates for visual processing speed (*C*) and threshold of conscious perception (*t*_0_) for all participants. Parameter estimates for letters and words were compared in paired-samples *t*-tests (see Table 1).

**Table 1:** **Performance and statistics across conditions for Experiments 1–3**.

**Parameter**	**Letters**		**Words**		**Statistics**			
	**Mean**	**(*SD*)**	**Mean**	**(*SD*)**	***t* (20)**	***P***	***r***	***d***
**EXPERIMENT 1**								
Reaction time	476	(38)	441	(46)	4.94	<0.001	0.74	0.78
**EXPERIMENT 2, SINGLE ITEM REPORT**								
*t*_0_	14.2	(7.1)	11.8	(3.3)	1.71	0.103	0.42	0.40
*C*	67.7	(24.1)	114.4	(40.4)	−5.50	<0.001	0.36	−1.36
**EXPERIMENT 3, WHOLE REPORT**								
*t*_0_	39.2	(12.9)	45.1	(18.7)	−1.53	0.142	0.42	−0.36
*C*	33.0	(15.6)	14.4	(7.3)	8.04	<0.001	0.81	1.07
*K*	3.9	(0.5)	2.5	(0.4)	13.19	<0.001	0.48	2.94
*w*_index_	0.72	(0.14)	0.60	(0.21)	2.96	<0.01	0.57	0.60

Units for individual parameters: Reaction time (ms), t_0_ (ms), C (items/s), K (items), and w_index_ (unitless).

### Experiment 3. whole report

Experiment 3 was designed to measure the participants' ability to perceive multiple independent stimuli simultaneously. Words and letters were presented in different blocks of 120 trials. There were four blocks in all. In every trial, six stimuli were chosen randomly without replacement from the stimulus sets described above. Stimuli were presented for one of six exposure durations (30–200 ms, randomly intermixed), and followed by either six pattern masks (500 ms), or a blank screen prolonging the effective exposure duration by a visual afterimage. Stimuli were shown at six locations on the circumference of an imaginary circle with a radius of 4.6° of visual angle centered on fixation(given this radius and the size of the words and letters used crowding effects between stimuli are minimal, see Kyllingsbæk et al., 2007). Again the instruction was to make unspeeded reports of the items which the subject was “fairly certain” of having seen, and responses were recorded by the experimenter. The first and second blocks for each stimulus type were preceded by 36 and 12 practice trials, respectively.

In the analysis, the raw scores (items correctly reported) for the different exposure durations were compared for the two stimulus conditions. Then, the performance of individual subjects was modeled by TVA (see section Mathematical Modeling for details) resulting in parameter estimates for speed of visual processing (*C*), threshold of conscious perception (*t*_0_), and capacity of VSTM (*K*), and attentional weights for each of the six stimulus positions. These weights were used to characterize any bias of attention toward the left or right visual hemifield by calculating a laterality index, *w*_index_, given as the ratio between the sum of the three weights in the left visual hemifield and the sum of all six attentional weights. This index ranges from zero (absolute right-sided bias) to one (absolute left-sided bias) with 0.5 indicating perfectly unbiased attentional weighting between the hemifields. An additional parameter was included to estimate the sensory decay in the unmasked trials; see Bundesen (1990). The mean estimates of *C*, *t*_0_, *K*, and *w*_index_ across subjects were compared for the letter and word conditions using paired-samples *t*-tests (see Table 1).

## Results

A summary of performance in the word and letter conditions in each experiment can be found in Table 1.

### Experiment 1. stimulus familiarisation

Mean RTs (SDs) were significantly longer for single letters, *M_LetterRT_* = 476 ms (37), than for words, *M_WordRT_* = 441 ms (45), see Table 1 for statistics. This difference was significant in 15/21 individual subjects. To be certain this was not attributable to the fact that there were more trials in the word condition, we also made this comparison with only the first 50 word trials. The RT advantage for naming words was slightly smaller when looking only at the first 50 word trials, *M*_50*WordRT*_ = 447 ms (48), but the difference was still highly significant, *t*_(20)_ = 3.75, *p* = 0.001.

### Experiment 2. single item report

**Figure 2:**
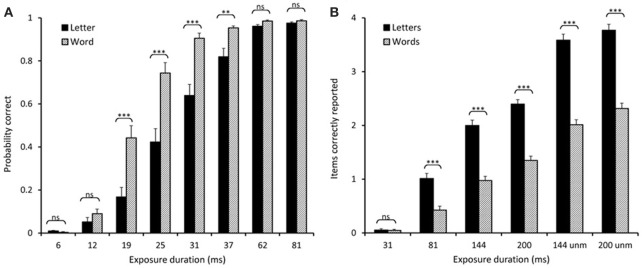
**Comparison of raw scores**. **(A)** Experiment 2: Proportion correct for letters and words at the different exposure durations. **(B)** Experiment 3: Number of items correctly reported for words/letters at the different exposure durations. ^**^p < 0.01, ^***^p < 0.001.

Figure 2, left panel, displays the raw scores (mean proportion of correct reports) for the two stimulus conditions at each exposure duration. Overall, words were identified significantly better than letters at all exposures from 19 to 37 ms. Participants were generally better at identifying words than letters, and significantly so in all conditions where floor effects (performance at exposures below the perceptual threshold) or ceiling effects (where performance were close to a 100% for both stimulus types) were not present. This difference was further qualified by the TVA-based parameter estimates.

A comparison of the mean TVA-estimates (across subjects) of *t*_0_ and *C* in the two conditions (see Table 1) revealed that the mean *t*_0_ values for letters (14.2 ms) and words (11.8 ms) were not significantly different. In contrast, the perceptual processing speed, the *C*-value, was significantly higher for words (114 items/s) than letters (68 items/s). This performance pattern is illustrated for a single, representative subject in Figure 1, left panel.

### Experiment 3. whole report

A comparison of the raw scores (items correctly reported, see Figure 2, right panel) showed that significantly more letters than words were reported at all exposure durations except for the shortest (30 ms), where performance in both conditions was close to zero. Indeed, the TVA-based modeling revealed that *t*_0_ was above 30 ms for both stimulus types, and not significantly different between letters and words (see Table 1). In contrast, processing speed (*C*) was significantly higher for letters (33.0 items/s) than words (14.4 items/s) in this experiment. In addition, the analysis revealed that significantly more letters (3.9 letters) than words (2.5 words) were retained in VSTM (*K*). See Figure 1, right panel, for an illustration of a single subject's performance and parameter estimates for the whole report of letters.

## General discussion

We investigated normal performance with single letters and short simple words in three experiments, aiming to explore the extents and limits of the WSE. In a naming task, we found that mean RTs were significantly shorter for words than letters. In our second experiment, single item report, we replicate the classical effect that words are identified better than letters with brief, masked presentation. Testing a range of stimulus durations, we found significantly better performance with single words than single letters at a several exposures between the perceptual threshold and ceiling performance.

In Experiments 2 and 3, we have adopted a novel approach to the investigation of the WSE by taking advantage of the TVA framework (Bundesen, 1990). This provides us with a more detailed picture of the factors underlying this effect, as we can derive several measures from one and the same task, and thus disentangle the contribution of e.g., perceptual processing speed and the threshold for perception. The combination of single item and whole report experiments further enables us to map out the perceptual process from the beginning of encoding the first word or letter, to the level where multiple word or letter representations are encoded in VSTM.

TVA-based modeling of data from Experiment 2 revealed that single words are processed significantly faster than letters, whereas the perceptual threshold did not differ between the two types of stimuli. In the third experiment, a classic whole report with multiple stimuli, a different pattern of performance emerged: Processing speed was faster for letters than words. Also, the capacity of VSTM, *K*, was significantly higher for letters than words.

### Extents and limits of the word superiority effect

Our findings indicate that the WSE is more general than previously reported. When presented in isolation, at the center of the visual field, single words are identified better than single letters at all exposure durations between the perceptual threshold and ceiling performance. The effect is also apparent in simple vocal reaction times to unmasked stimuli, perhaps indicating that words enjoy “superiority” not only at perceptual levels of processing. However, although single words are perceived and reported better and faster than single letters, words do not enjoy the same advantage when multiple stimuli are presented simultaneously. In such cases, single letters are processed faster than words, and, in addition, more single letters than whole words can be encoded into VSTM. Also, there is a general decrease in processing speed for both stimulus types from the single item to the whole report experiment. It is well-known that both errors and RTs increase with eccentricity (Eriksen and Schultz, 1977; Carrasco et al., 1995), and thus this speed dependence on eccentricity is not unexpected.

The WSE has typically been reported in experiments using brief, masked displays of single stimuli (e.g., McClelland and Johnston, 1977), and forced choice reponses. The type of masking or degradation required to evoke this effect has been widely debated (Johnston, 1981; Prinzmetal, 1992; Jordan and Bevan, 1994), and most studies have used the one exposure duration where subjects perform about 75% correctly (Pollatsek and Rayner, 2005). Our results suggest that this may not be necessary, as the WSE, at least when measured with a report task rather than forced choice, is evident over a range of exposure durations. Using a two-alternative forced-choice paradigm comparing performance with postmasked words and single letters, Jordan and de Bruijn(1993; see also Jordan and Bevan, 1994) found that word superiority persisted only when the same size masks were used for both words and letters but disappeared when the width of the masks were adjusted to the actual width of the individual stimuli. This latter approach, however, may inadvertently have resulted in a letter benefit as certain letters could easily be excluded just by the size of their masks. Hence, we used similar masks for both letters and words. Even if the WSE we observed in Experiment 2 could potentially be explained by the mask we used, this does not necessarily make the effect less interesting. Also, mask attributes cannot explain why the effect is reversed in Experiment 3, where the same stimuli and masks were employed.

In addition, the results of Experiment 1 indicate that words are processed more efficiently than single letters even when they are unmasked. Cattell (1886) was the first to record such a word superiority in vocal naming times, but the phenomenon has not been studied to any large degree, although it does, in our opinion, deserve further investigation. For instance, it is possible that some of the word advantage in RTs may have its roots on other levels of processing than in visual perception, and may perhaps be related to the ease of phonological retrieval. The relative speed of lexical and sublexical processing has been investigated within the framework of the Dual Route Cascaded model of reading (Coltheart et al., 2001). Sublexical processing (letter-sound translation processes) is slower than lexical (whole word) processing, and this may be related to the RT difference we observe between single letters and words. It may also be the case, however, that the advantage in visual processing speed observed for words compared to letters in Experiment 2 contributes to the overall difference in RT, and this would be interesting to investigate further.

One question that remains is why words—when they are so effectively processed alone—do not enjoy the same advantage when multiple stimuli are presented simultaneously. Why can our subjects not encode as many words into their VSTM as they can letters? First, this argues against the notion that words are processed as units, or at least as units encodable in VSTM. Similar to the 7 ± 2 rule for verbal short term memory (Miller, 1956), VSTM is known to have a capacity of about four items (Sperling, 1960). This is qualified by the finding that capacity decreases as objects become more complex (Alvarez and Cavanagh, 2004), which could perhaps explain our finding, as words are obviously more visually complex than single letters. On the other hand, some studies indicate that VSTM capacity is larger for objects of expertise than unfamiliar objects (Curby et al., 2009). Being fluent readers, our subjects are indeed experts in word identification, and in that light the limit of their VSTM capacity for words seems surprisingly low. Another possible explanation of the reversed effect in the whole report experiment is that stimuli were presented outside the central visual field (at 4.6° from fixation). Although previous work indicates that there is little crowding between stimuli at this eccentricity Kyllingsbæk et al., 2007), “within stimulus crowding” (i.e., lateral masking) may have affected the processing of words in this condition. Jordan and Patching (2004) have shown that the word-letter phenomenon can be reversed when stimuli are presented in lateralized displays, which resembles the effect we find in Experiment 3. They suggest that while crowding effects (or effects of lateral masking) are counteracted by strong lexical activations when words are presented foveally, such top–down effects do not prevent crowding in lateralized displays. This presents a challenge for our ability to measure the capacity of VSTM for word stimuli, however, as it will be difficult to avoid both within and between stimulus crowding in the same paradigm, while keeping stimuli in the central visual field.

It is worth noticing, however, that if we count the number of letters encoded in the word condition in Experiment 3, we do see a WSE: While our subjects could only encode a mean of 2.5 (i.e., 2 or 3) three-letter words at the longest exposure durations, this of course translates to them having encoded between six and nine *letters*. This is clearly superior to their performance in the letter condition, where the mean capacity was about four letters. Thus, the WSE may be said to be present also in the whole report condition, but not to the same extent as in the single item task.

### Future directions

We have previously used methods based on TVA to investigate visual processing in the disorder of pure alexia, where word reading is disrupted by brain injury, typically affecting the visual word form area and surrounding structures (Starrfelt et al., 2009, 2010). We have shown that this seemingly selective reading disorder is characterized by reduced central processing speed not only for letters but also for digits, and reduced VSTM capacity for both types of stimuli. An interesting extension of the current work would be to compare pure alexia patients' performance with words and letters using similar paradigms. The reading deficit in pure alexia affects both word and letter identification, but yet a WSE (words vs. non-words) has been reported in some patients with this disorder (Behrmann et al., 1998). Indeed, in the same patients where we observed reduced central processing speed and VSTM capacity for unrelated letters and digits, we also found better report of letters from words compared with non-words (Starrfelt et al., 2013), although the WSE was generally smaller in patients than in controls. The word-letter experiments presented in the current paper seem fit to characterize the relationship between letter and word processing in pure alexia further. Pure alexia is thought to be a deficit in parallel processing of letters, resulting in a compensating strategy of serial letter identifications (and thus a large effect of word length on reading times). If this is the case, we should expect patients to show the opposite pattern of performance in our single stimulus word-letter experiments compared to normal subjects: they should be slower in naming words than letters, and show reduced processing speed for words compared with letters. Indeed, if pure alexia truly abolishes parallel letter processing, one would expect their threshold for identifying three letter words to be three times as high as for single letters.

## Conclusion

We have shown that the WSE, at least for simple short words, can be revealed in vocal reaction times, and that part of this superiority is probably caused by increased visual processing speed for words compared to letters. This fits neatly with previous observations of the WSE, and the interpretation that top–down connections may enhance processing of letters in words, while single letter processing may rely more on bottom-up signals. A novel finding is that the WSE is significant at a range of exposure durations, which means that at least in our paradigm, the meticulous search for a given performance level is not necessary to reveal the effect. Rather, words seem to be processed better or faster than letters from the threshold of perception. When several stimuli are presented simultaneously, we find the opposite result: letters are processed faster than words, and more letters than words can be encoded in VSTM. This indicates that words are not treated as units in VSTM.

### Conflict of interest statement

The authors declare that the research was conducted in the absence of any commercial or financial relationships that could be construed as a potential conflict of interest.

## Appendix

### Word stimuli

All words are high frequency Danish words, with high neighborhood-size. At least two neighbor words were included in the list for all stimuli, thus making it necessary to process at least two, and for most words all three letters in the word to identify it correctly.

**Table:**

**Stimulus**	**Freq. pr mill.^a^**	***N*_size^b^**	**Wordclass**	**Neighbour-stimuli in list**	
bag	266	25	Noun; prep	3	bog; dag; tag
bog	107	23	Noun	2	bag; tog
dag	791	23	Noun	3	bag; dig; tag
den	9259	28	Det.; pron.	2	det; din
det	15358	22	Det.; pron.	2	den; dit
dig	427	21	Pron.	4	dag; din; dit; mig
din	267	24	Pron.	4	den; dig; dit; min
dit	111	24	Pron.	3	det; dig; din
fad	23	19	Noun	3	far; fod; mad
far	212	24	Noun	2	fad; for
fod	29	18	Noun	3	fad; for; mod
for	9336	22	Conj.	3	far; fod; mor
han	4556	21	Pron.	3	hun; kan; man
hun	2070	17	Pron.	2	han; kun
kan	4058	15	Verb	3	han; kun; man
kun	970	14	Adv.	2	hun; kan
mad	85	18	Noun	4	fad; man; med; mod
man	3146	17	Pron.; noun	4	han; kan; mad; min;
med	9204	15	Prep.; adv.	2	mad; mod
mig	1123	18	Pron.	2	dig; min
min	684	20	Pron.	3	din; man; mig
mod	907	16	Noun; prep	4	fod; mad; med; mor
mor	244	19	Noun	2	for; mod
tag	78	22	Noun; verb	3	bag; dag; tog
tog	290	15	Noun	2	bog; tag
Mean	2544.04	20		2.8	
SD	4019.46	3.70		0.76	
Median	684	4		2	

a Bergenholtz (1992).

b Number of words in the Danish dictionary (www.ordnet.dk/ddo) differing from the target by only one letter. Values kindly calculated by the Danish Lexicographic Society.
